# Influence of New Compound Disinfectant From N-Dodecyl-2-(Piridin-1-Ium)Acetamide Chloride on Pathogenic Microorganisms in Poultry Houses

**DOI:** 10.3389/fmicb.2021.735859

**Published:** 2021-09-24

**Authors:** Nannan Chen, Pingwei Qin, Yu Liu, Ying Yang, Hairuo Wen, Lihua Jia, Jing Li, Zhanbo Zhu

**Affiliations:** ^1^College of Animal Science and Veterinary Medicine, HeiLongJiang BaYi Agricultural University, Daqing, China; ^2^Microbiology Research Laboratory, Branch of Animal Husbandry and Veterinary of Heilongjiang Academy of Agricultural Sciences, Qiqihar, China; ^3^National Center for Safety Evaluation of Drugs, Key Laboratory of Beijing for Nonclinical Safety Evaluation Research of Drugs, National Institutes for Food and Drug Control, Beijing, China; ^4^Key Laboratory of Fine Chemicals of College of Heilongjiang Province, College of Chemistry and Chemical Engineering, Qiqihar University, Qiqihar, China

**Keywords:** quaternary ammonium salt, antibacterial activity, synergistic effect, safety, microorganism

## Abstract

With the development of large-scale and intensive poultry farming, environmental disinfection has become particularly important, and the effectiveness of disinfection depends upon the performance of the disinfectants. Quaternate ammonium salt is a group of positively charged polyatomic ions with both antibacterial and antiviral activities. In order to prepare an ideal disinfectant for poultry farms, we combined a quaternate ammonium salt *N*-dodecyl-2-(piridin-1-ium)acetamide chloride with two other disinfectants (chlorhexidine acetate and glutaraldehyde), respectively. The antimicrobial activity, mutagenicity, and safety of the compound disinfectants were assessed by the European Standard methods using ATCC strains and clinical isolates. The results showed that both compound disinfectants meet the requirements of microbial reduction, and their effectiveness was not affected by organic matter. Quaternary ammonium disinfectant resistance genes were not detected in the strains tested indicating that bacteria are less likely to develop resistance to these compound disinfectants. Ames test showed that there was no detectable mutagenicity in the strains treated with the compound disinfectants. *In vivo* experiment showed that both compound disinfectants did not have significant pathological effect in mice. The bactericidal effect of the compound disinfectants was not significantly different among strains of different sources (*p*>0.05). Clinical tests showed that compound disinfectant had a good bactericidal effect on the air and ground of poultry farms. These results show that quaternary ammonium salts in combination with other compounds can enhance the bactericidal effect and can be used safely in poultry feedlots. This study provides a technical reference for the development of a new quaternate ammonium compound disinfectant with strong disinfection effect and low irritation.

## Introduction

Microbial infections are one of the most harmful diseases to the poultry industry (Ho et al., 2010; Medzhitov et al., 2012). They not only cause an increase in mortality and the economic loss of poultry products, but also pose a serious threat to human health. Disinfectants play an important role in reducing the occurrence of infectious diseases that affect the development of the poultry industry. At present, disinfectants commonly used in poultry farms include peroxyacetic acid, sodium hydroxide, povidone-iodine, and quick limes. However, these disinfectants are prone to produce resistance and drug residues after long-term use, and some of them also have the problems of causing irritation and corrosion. Therefore, it is important to develop new broad-spectrum disinfectants.

Quaternary ammonium salt disinfectant plays a vital role in the prevention of animal diseases (Ioannou and Hanlon, 2007; Murguía et al., 2008; Giardino et al., 2016; Zhang et al., 2016; Song et al., 2018). Previous studies have shown that the combination of a quaternary ammonium salt with chlorhexidine gluconate produces a synergistic effect. Chlorhexidine gluconate is one of the most widely used antimicrobial agents because it has broad-spectrum antibacterial properties and is compatible with many kinds of materials (Sandle, 2019). It is often used as a compound or monomer disinfectant (Hidalgo and Dominguez, 2001; Bhende and Spangler, 2004; Chapman et al., 2012). Cationic surfactants also produce synergistic fungicidal effects by combining with nitrogen moiety, pyridine, or quacking (Brandes et al., 1993). Studies have shown that quaternate ammonium salts are vulnerable to organic matter and usually fail when they are used alone. Therefore, the combination of 25% glutaraldehyde, 25% double-chain quaternate ammonium salt, and ethanol not only enhanced the stability but also expanded the bactericidal spectrum (Martin, 1994). Combination of 0.1% dimethyl ammonium chloride, 0.1% methylammonium bromide, and 0.2% isopropanol can kill *Salmonella* ATCC 10708 and *Staphylococcus aureus* ATCC 6538 within 20min (Wachman and Karlan, 1990). These studies demonstrated that compound of quaternate ammonium surfactant had broad development and application prospects. In this study, we combined a quaternate ammonium surfactant with other disinfectants and determined their bactericidal activity and clinical applications. Our results indicated that this new compound disinfectant has high bactericidal effect and could be used safely in poultry feedlots.

## Materials and Methods

### Reagents and Strains

Experimental animals include 40 SPF Kunming mice (18–22g). Animals were purchased from the Harbin Medical University. A total of 30 chicks of Hy-line variety brown at 1day of age were provided by the Harbin Yinong Poultry Industry Co. The clinical trial was completed in mid-January at a poultry farm in Longjiang County, Qiqihar, where sampling was completed. Quaternary ammonium salt cationic surfactant was synthesized by Professor Lihua Jia of Qiqihar University (Table 1).

**Table 1:** Cationic surfactant structural formula.

Compound structural formula	Naming of compounds	Abbreviations	Source of compounds
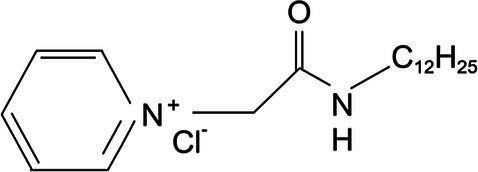	*N*-dodecyl-2-(piridin-1-ium)acetamide chloride	C_12_cmpCl	Zhuo Feng 2015 (*Res. Chem.Intermed*)
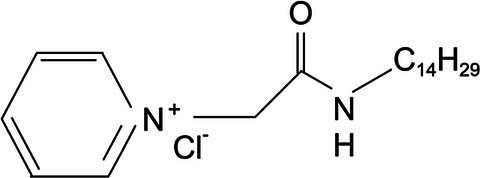	*N*-tetradecyl-2-(piridin-1-ium)acetamide chloride	C_14_cmpCl	Zhuo Feng 2015 (*Res. Chem.Intermed*)
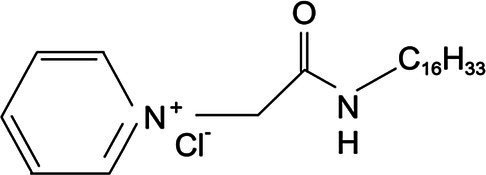	*N*-cetyl-2-(piridin-1-ium)acetamide chloride	C_16_cmpCl	Zhuo Feng 2015 (*Res. Chem.Intermed*)

*C_12_cmpCl, N-dodecyl-2-(piridin-1-ium)acetamide chloride; C_14_cmpCl, N-tetradecyl-2-(piridin-1-ium)acetamide chloride; and C_16_cmpCl, N-tetradecyl-2-(piridin-1-ium)acetamide chloride*.

ATCC strains and isolates from poultry farm were stored at the Heilongjiang Provincial Engineering Technology Research Center for Prevention and Control of Cattle Diseases (Table 2).

**Table 2:** ATCC standard strains for experiments.

Species	Acronym	Strain number	Source
*Pseudomonas aeruginosa*	*P. aeruginosa*	ATCC 15442	SHBCC
*Staphylococcus aureus*	*S. aureus*	ATCC 6538	SHBCC
*Escherichia coli*	*E. coli*	ATCC 10536	SBBTCL
*Bacillus subtilis*	*B. subtilis*	ATCC 6633	SHBCC
*Enterococcus hirae*	*E. hirae*	ATCC 10541	SBBTCL
*Proteus vulgaris*	*P. vulgaris*	ATCC 13315	SBBTCL
*Candida albicans*	*C. albicans*	ATCC 10231	SHBCC
*Aspergillus brasiliensis*	*A. brasiliensis*	ATCC 16404	SHBCC

*SHBCC, Shanghai Bioresource Collection Center; SBBTCL, Shanghai Beinuo Biological Technology Co., Ltd*.

*Salmonella typhimurium* histidine nutrient-deficient (*his^−^*) strains TA97, TA98, TA100, TA1535, and TA1537 were obtained from the Japanese (strain) Bioscience Center (JBSINC). The strains were isolated and screened for the presence of amino acid and biotin synthesis defects, cell wall lipopolysaccharide deficiency (*rfa*), UVR (*uvrA* or *ΔuvrB*) repair defects, and resistance to ampicillin and tetracycline (containing pKM101 or pAQ1 plasmid). The strains meeting the requirement were used in this study. The positive control reagents (2-2-Furyl-3-5-Nitro-2-Furylacrylamide, AF-2 and 2-Aminoanthracene, 2-AA) were purchased from Sigma. Sodium azide (NaN_3_) was purchased from Merck. 9-aminoacridine and 9-AA were purchased from Acros Organics. CM0067 nutrient broth No.2 was purchased from OXOID. Agar powder, ampicillin, glucose, histidine, tryptophan, biotin, MgSO_4_·(7H_2_O), sodium citrate·(2H_2_O), K_2_HPO_4_·(3H_2_O), KH_2_PO_4_, (NH_4_)_2_SO_4_, NADP, G-6-P, KCl, and MgCl_2_ were purchased from Sigma. Rat liver S9 mixture (made from the combination of sodium phenobarbital and β-naphthoflavone-induced rat liver) was purchased from the Beijing Kangruijie Technology Co. Ethanol (Cat. No. 200802, Quanrui Reagent Co.), chlorhexidine acetate (Cat. NO. 190104, Jiu Tai Reagent Company), Glutaraldehyde (Cat. NO. 190417 Tianjin Hongyan Reagent Factory), and Bovine serum albumin (BSA; Cat. NO. EZ2921B398, BIOFROX) were used in this study.

### Preparation of Quaternary Ammonium Salt Compound

The pure 1-(alkylcarbamoylmethyl) pyridinium chloride was described as C_n_cmpCl, where n (= 12, 14, 16, respectively) represents the carbon number of alkyl. In this study, C_12_cmpCl, C_14_cmpCl, and C_16_cmpCl were used (Table 1). The FT-IR (BK_r_), ^1^HNMR, and ESI–MS (*m/z*) data of the C_12_cmpCl were shown below. The compound was obtained in 91% yield with M.p of 118.8–119.5°C. FT-IR spectrum (KBr) *ν*_max_: 3399.32, 3207.26, 3056.07, 2953.92, 2917.14, 2851.76, 1666.74, 1638.14, 1568.67, 1491.03, 1466.52, 788.20, 726.90, 706.47, and 677.87cm^−1^. ^1^H–NMR (CDCl_3_, 400MHz): *δ* 0.88 (t, *J* =6.8Hz, 3 H, C*H*_3_), 1.28 (m, 18 H, CH_3_–(C*H*_2_)_9_), 1.58 (m, 2 H, C*H*_2_–CH_2_–NH), 3.22 (m, 2 H, C*H*_2_–NH), 5.98 (s, 2 H, CO–C*H*_2_), 8.04 (t, *J* =7.4Hz, 2 H), 8.46 (t, *J* =7.8Hz, 1 H,), 9.44 (d, *J* =5.2Hz, 2 H), and 9.50 (t, *J* =2.4Hz, 1 H, N*H*–CO) ppm. MS (ESI) *m/z* was [M–Cl^−^]^+^ Calcd: 305.3, Found 305.3 (Feng et al., 2015).

Chlorhexidine acetate was dissolved in alcohol and mixed with C_12_cmpCl at a ratio of 2:1 (ethanol 10%). This quaternary ammonium salt composition was designated as compound disinfectant-1 (hereinafter referred to as C_1_). The second compound was composed of glutaraldehyde and C_12_cmpCl at a ratio of 2:1 (hereinafter referred to as C_2_). The final concentration of C_12_cmpCl is 0.7 (g/l) in C_1_ and C_2_. The final concentration of chlorhexidine and glutaraldehyde is 1.4 (g/l) in C_1_ and C_2_, respectively. Disinfectant dilutions were prepared in water of standardized hardness (WSH) immediately before testing (VAH, 2015).

### Antimicrobial Activity Assay

#### Neutralization Test

Neutralization test was conducted according to phase 1 tests (EN 1040 and EN 1275) and then according to phase 2, step 1 tests (Draft EN 13727 and EN 13624; EN 13624, 2003; EN 1040, 2005; EN 1275, 2005; CEN, 2015; EN 13727, 2015). PBS containing 0.5% w/v glycine, 0.5% w/v lecithin, and 1% v/v Tween 80 was used as neutralizer in the tests.

#### Minimum Inhibition Concentration Test

Minimum inhibition concentrations of disinfectants were determined by the microdilution method as described by NCCLS, VAH method 7 and EN 14885 (Cockerill, 2000; EN 14885, 2015; VAH, 2015). C_12_cmpCl were used as the monomer active substances for bactericidal activity tests. Tests were performed using 96-well cell culture plates. Dilute the compound disinfectant stock solution to different concentrations. The concentrations of the compound disinfectants ranged from 0.1 to 1,000mg/l. In the experimental group, 80μl nutrient broth medium, 10μl compound disinfectant, and 10μl bacterial suspension were added to each well. For the blank control, only nutrient broth medium was added. For the positive control, microorganisms and nutrient broth medium were added. The monomer compound disinfectant was added and used as the monomer control. Subsequently, the culture was incubated at 37°C for 24h.

#### Minimum Bactericidal Concentration Test

The minimum bactericidal concentration (MBC) test was measured according to the Deutsche Gesellschaft for Hygiene, Microbiologic (DGHH) method and EN 14885 (Begec et al., 2013; EN 14885, 2015; VAH, 2015), and 100μl was added to nutrient agar solid medium and cultured in 37°C incubator for 24h. MBC was judged by the presence of colonies. This measurement was performed twice. Bacterial test in suspensions contained 1.0–5.0×10^8^ colony-forming units (CFU)/ml, except for the practical tests on steel carriers which contained 1.0–5.0×10^9^CFU/ml.

#### Time Kill Assay

With reference to VAH method 8 and EN 14885 (EN 14885, 2015; VAH, 2015), the compound disinfectants were dissolved in hard water at different dilutions. The test disinfectant solution and the test bacteria were mixed together in suspension and incubated in a 20°C water bath at a constant temperature. Under this condition, four time points (1, 5, 15, and 30min) were selected to determine the killing of the indicator bacteria. Test bacteria suspension (0.1ml) and 10ml of disinfectants were added into a sterile test tube, and mix immediately for four time points. Subsequently, 0.1ml of sample solution was added to a test tube containing 10ml of neutralizer and mixed for 10min. Then, 0.1ml was added to a 10ml nutrient broth tube. After incubating at 37°C for 24h, bacterial growth was evaluated visually. Quantitative test in suspension was performed as described above. After 10min of neutralization, culture was incubated on nutrient medium at 37°C for 24h to enumerate the viable bacteria. Each experiment was repeated for three times.

#### Organic Substance Protection Test

In order to further evaluate the bactericidal activity of the disinfectants, we used Vah Method 9, EN13727, and EN 14885 (EN 13727, 2005; EN 14885, 2015; VAH, 2015). The organic substance protection test was carried out at 20°C. Bovine serum albumin was selected as an organic substance (3g/l BSA). Among the recommended action time, 1, 5, 15, and 30min were selected. Briefly, the disinfectant solution (8ml) was mixed with BSA (1ml), and 1ml of bacterial suspension was then added. After mixing, it was allowed to grow for four different time points, and then, 0.5ml was transferred to 4.5ml of neutralizer and mixed for 10min. Subsequently, 100μl was spread on nutrient agar medium and incubated at 37°C for 48h for colony enumeration.

### Carrier Test

Biocide efficacy under practical conditions with organic load (i.e., surface disinfection without mechanical action; VAH method 14.1 and BS EN 13697, 2019) was evaluated using stainless steel carriers (VAH, 2015). A 50-μl drop of bacterial suspension was dried (60min at 37°C) on stainless steel carriers (1cm diameter) and was covered with 100μl of disinfectant for the exposure times suggested by VAH guidelines (i.e., 1, 5, 15, or 30min at 20°C). Subsequently, carriers were transferred to 10ml of neutralizer. Finally, biocide efficacy was determined by colony enumeration.

### Detection of Quaternary Ammonium Salt Resistance Genes

In order to determine the potential resistance of bacteria to disinfectants, five pairs of primers specific for disinfectant resistance-related genes were designed. Qac E∆1, qac A/B, qac C, qac G, and qac J are all quaternary ammonium disinfectant resistance genes (Rajamohan and Srinivasan, 2010; Table 3). PCR was performed to determine the presence of these genes in bacteria. PCR was performed with the follow conditions: initial denaturation at 93°C for 2min, followed by 35cycles of 93°C for 30s, 56°C for 30s, extension at 72°C for 1min, and a final extension at 72°C for 5min (for amplification of *qacEΔ1*); initial denaturation at 93°C for 2min, followed by 35cycles of 93°C for 30s, 56°C for 30s, extension at 72°C for 1min, and a final extension at 72°C for 5min (for amplification of *qacA/B* and *qacC*:); initial denaturation at 93°C for 2min, followed by 35cycles of 93°C for 30s, 48°C for 30s, extension at 72°C for 1min, and a final extension was performed at 72°C for 5min (for amplification of *qacG* and *qacJ*).

**Table 3:** Primer sequences for amplification of quaternary ammonium resistance genes.

Target gene	Primer sequence (5'-3')	PCR product (bp)
*qacEΔ1*	F: TAGCGAGGGCTTTACTAAGC	300
	R: ATTCAGAATGCCGAACACCG	
*qacA/B*	F: TCCTTTTAATGCTGGCTTATACC	220
	R: AGCCATACCTGCTCCAACTA	
*qacC*	F: GGCTTTTCAAAATTTATACCATCCT	249
	R: ATGCGATGTTCCGAAAATGT	
*qacG*	F: TACATTTAAGAGCACTACA	242
	R: CATCCAAAAACGTTAAGA	
*qacJ*	F: CTTATATTTAGTAATAGC	239
	R: CATCCAAAAACGTTAAGA	

### Bacterial Reverse Mutation (Ames) Test

The Ames test was conducted in accordance with OECD Guideline 471 (2020) and Díez-Quijada et al. (2019). *S. typhimurium* histidine nutrient-deficient (*his^−^*) strains TA97, TA98, TA100, TA1535, and TA1537 were used as the indicator bacteria in the test. The compound disinfectant was first determined not to inhibit *S. typhimurium* at the highest concentration before subsequent experiments were performed. Briefly, the bacterial freeze–thaw solution was mixed with nutrient broth and then incubated in a water bath shaker at 37°C and 120r/min for 10h. After amplification, the optical density was measured and the concentration of viable bacteria was estimated to be 1×10^9^/ml for subsequence test. Positive controls included 2-(2-furyl)-3-(5-nitro-2-furyl)acrylamide (AF-2), 2-Aminoanthracene (2-AA), sodium azide, NaN_3,_ 9-aminoacridine (9-AA). Negative control with addition of solvent only was also included. Five concentrations were established according to subject inhibition, and Ames tests were carried out using 6-well plates in the presence/absence of S_9_ metabolic activation. All samples were incubated at 37°C for approximately 48h, and then, the colonies were enumerated. The experiment was repeated twice. The number of responding colonies in each well was enumerated, and the mean value was obtained separately for each concentration group and expressed as mean standard deviation (±). The average number of revertant colonies per dish/well in the TA1535 and TA1537 test groups was three times higher than the control group, while the average number of revertant colonies per dish/well in the other strains was 2 times higher than the solvent control group and a quantitative relationship was found to be positive; otherwise, the result was considered negative.

### Toxicological Evaluation

#### Acute Oral Toxicity Test

The acute oral toxicity test was conducted according to the OECD (OECD, 2008; Alluri et al., 2019). Food was given for 3.5h after administration using the one-time maximum limit method. The dose was 5,000mg/kg body weight, and the dose was administered by oral gavage at 20ml/kg/BW. Poisoning performance was recorded. Weight of test animals, and autopsy of dead and expired animals were performed. Tissue or organ abnormalities were analyzed by histopathological examination.

#### Immunohistochemistry Test

Immunohistochemistry test was performed as described previously (Xue et al., 2018). Animals were divided into high-dose group, middle-dose group, low-dose group, and control group. Gastric perfusion was performed once a day; the duration of the test was 7, 14, 30 days, and 24h after the last exposure. Euthanasia was performed with cervical dislocation under deep anesthesia. Tissue specimens were fixed in 10% formalin buffer, dehydrated, removed, and paraffin-embedded. Routine histological staining of 5-μm sections was performed with hematoxylin and eosin (H and E).

#### DNA Damage Detection in Poultry

The test started with determining the LD_50_ in the chicks. Acute oral toxicity test for chicks was conducted according to OECD method (OECD 205, 1984; OECD 423, 2001; OECD 223, 2016). The compounds were administered by oral gavage at the dose of 2000mg (in 20ml)/kg body weight. Clinical signs and mortality of animals were recorded after oral administration, and LD_50_ was calculated. Based on LD_50_, DNA damage test was performed in chicks. One-day-old chicks were fed regularly for 1week. Chicks were randomly divided into five groups (six chicks in each group according to body weight, with a 50/50 split between males and females): group of combined disinfectant (C_1_/C_2_), highest-dose group (2000mg/kg), monomeric compound groups (C_12_cmpCl), negative control group (saline), and positive control group (Ethyl methanesulfonate). The animals were maintained at a constant temperature of 22°C (±3°C) and humidity of approximately 70% throughout the study. After 1week of acclimatization, animals were inoculated by a single gavage at the dose of 2000mg (in 20ml)/kg body weight after 2h of fasting. Animals were monitored for clinical signs and mortality every 2h after administration. If no signs of poisoning or mortality occur after 7days, the experiment was extended to 14days before dissection. Cells were extracted from liver and spleen and thymus tissues, respectively, and lymphocyte DNA damage was determined by single-cell gel electrophoresis (SCGE). The SCGE assay was performed according to the OECD method (OECD TG489, 2014) and Kassie et al., 2002. DNA damage was determined by measuring the length of the comet tail of the cells with a fluorescent microscope.

### Determination of Bactericidal Effect on Strains From Different Sources

In order to evaluate the antibacterial stability of the compound disinfectant, we selected *Escherichia coli*, *Salmonella enteritidis*, *S. aureus*, *Streptococcus pneumoniae*, *Pasteurella multocida*, and *Bacillus subtilise* from different sources for evaluation. Based on the results of the carrier quantification test, the logarithmic values of the pathogenic bacteria killed by the compound disinfectants were recorded in different time points (5, 10, 15, and 20min) by selecting the appropriate concentrations. Each group selects 10 strains from diverse sources for MBC test. Multiple comparative analyses were performed using SPSS 19.0 software.

### Poultry Farms Disinfection Test of Compound Disinfectant

#### Air Disinfection Test

Air disinfection test was performed as described previously (VAH, 2015). Windows and ventilation holes were cleaned and closed before disinfection. The two kinds of compound disinfectants were selected from the range of 3.90–500mg/l according to the results from previous experiments. Disinfectants were sprayed into the air in the house, and the flat-plate landing method was used to collect samples before and 10, 20, 40, and 60min after disinfection. Samples were placed into an incubator for 24–48h, and colonies were enumerated.

#### Disinfection Test in the Floor of Poultry Farms

First, the disinfectant was diluted with WSH at different concentrations. In the poultry house, three areas of l×lm^2^ on the ground were selected, and the dirt on the ground was removed. Subsequently, diluted disinfectants with different concentrations were used for disinfection. During disinfection, we selected four areas along a diagonal line (each with an area of 10×10cm^2^) and collected samples before disinfection and 10, 20, 40, and 60min after disinfection. Sterilized cotton swabs were dipped in the collected samples. The neutralizer was taken and rubbed it on the collected area before being placed into a sterile test tube (5ml/tube) containing the neutralizer. The collected samples were brought back to the laboratory, the bacteria on the cotton swab were fully eluted, and then, the bacterial suspension was diluted 10 times with sterile normal saline. Finally, the samples were incubated in a 37°C incubator for 12–24h for colony enumeration.

## Results

### Antibacterial and Antifungal Activities of Disinfectants

#### Screening of Optimal Surfactants

PBS containing 0.5% w/v glycine, 0.5% w/v lecithin, and 1% v/v Tween 80 was used as neutralizer in the tests. Contact time was 10min at 20°C for the disinfectant-neutralizer mixture. A control group containing sterile distilled water was used to verify that the neutralizing agent had no bactericidal effect. The control group showed that the neutralizing agent had no antibacterial activity and the neutralizing agent and neutralization product had no effect on the growth of the indicator bacteria.

A series of surfactants were then screened, and the surfactant with the best bacterial inhibition effect was selected for compounding studies based on the minimum inhibition concentration (MIC) test results. Bactericidal activity of the compound disinfectant was determined with the monastic compound as a control. Among the series of cationic surfactants, C_12_cmpCl exhibited the best antibacterial and antifungal effect (*p* <0.001). C_12_cmpCl showed the same inhibitory activity against *S. aureus, E. hirae, E. coli*, and *B. subtilis*, with a minimum inhibitory concentration of 31.25mg/l for all of these organisms. The inhibition criteria for *Candida albicans* and *Aspergillus brasiliensis* were met when the concentration was increased to 62.50mg/l. The inhibitory concentration against *Pseudomonas aeruginosa* and *Proteus vulgaris* was 125mg/l (Table 4). Both C_14_cmpCl and C_16_cmpCl did not exhibit as good inhibitory activity as C_12_cmpCl. Based on these results, we selected C_12_cmpCl for the subsequent studies.

**Table 4:** Determination of the minimum inhibitory concentration of a series of cationic surfactants.

Microorganism	Minimum inhibitory concentration (mg/l)		
	C_12_cmpCl	C_14_cmpCl	C_16_cmpCl
*P. aeruginosa*	125^A^	500^B^	500^B^
*S. aureus*	31.25^A^	250^C^	125^B^
*E. hirae*	31.25^A^	500^C^	250^D^
*E. coli*	31.25^A^	500^C^	500^C^
*P. vulgaris*	125^A^	500^C^	500^C^
*C. albicans*	62.50^A^	500^B^	500^B^
*A. brasiliensis*	62.50^A^	500^C^	500^C^
*B. subtilis*	31.25^A^	500^C^	250^D^

*Different uppercase letters in horizontal data indicate extremely significant difference (p<0.001). Identical letters or no shoulder marks indicate insignificant difference (p>0.05). C_12_cmpCl, N-dodecyl-2-(piridin-1-ium)acetamide chloride; C_14_cmpCl, N-tetradecyl-2-(piridin-1-ium)acetamide chloride; C_16_cmpCl, N-tetradecyl-2-(piridin-1-ium)acetamide chloride; P. aeruginosa, Pseudomonas aeruginosa ATCC 15442; S. aureus, Staphylococcus aureus ATCC 6538; E. hirae, Enterococcus hirae ATCC 10541; E. coli, Escherichia coli ATCC 10536; P. vulgaris, Proteus vulgaris ATCC 13315; C. albicans, Candida albicans ATCC 10231; A. brasiliensis, Aspergillus brasiliensis ATCC 16404; and B. subtilis, Bacillus subtilis ATCC 6633*.

#### MIC and MBC of Disinfectants

In the time kill assay, the antibacterial and antifungal effect of the compound disinfectant was significantly higher than that of the monomer component (*p* <0.001). The bactericidal effect of the compound disinfectant C_1_ was superior to that of C_2_, with a MBC of only 3.90mg/l against *S. aureus* within a 1min action time. The MBC of C_2_ against *S. aureus* was approximately four times higher than that of C_1._ The compound disinfectant C_1_ showed the same bactericidal and fungicidal activity against *E. hirae*, *E. coli*, *C. albicans*, *A. brasiliensis*, and *B. subtilis*. Its bactericidal activity against *P. vulgaris* and *P. aeruginosa* was slightly lower with the bactericidal concentration of 15.62mg/l and 31.25mg/l, respectively. The MBC of C_2_ against *P. vulgaris* and *P. aeruginosa* was 62.50mg/l (Table 5). These results showed that MIC and MBC of C_1_ were lower than those of C_2_, indicating that antibacterial (bacteriostatic and bactericidal) effect of C_1_ is better than that of C_2_. Furthermore, C_1_ had a more significant bacteriostatic and bactericidal effect on Gram-positive bacteria than that on Gram-negative bacteria.

**Table 5:** Bactericidal concentrations resulting in a ≥5 logarithmic reduction in the quantitative suspension test.

Microorganism	C_1_ (mg/L)				C_2_ (mg/L)				Monomer (mg/L)			
	1min	5min	15min	30min	1min	5min	15min	30min	1min	5min	15min	30min
*P. aeruginosa*	31.25	31.25	15.62	15.62	62.50	62.50	31.25	31.25	250	250	125	125
*S. aureus*	3.90	1.95	1.95	0.97	15.62	15.62	7.81	7.81	62.50	62.50	31.25	31.25
*E. hirae*	7.81	7.81	3.90	3.90	15.62	15.62	7.81	7.81	62.50	62.50	31.25	31.25
*E. coli*	7.81	7.81	3.90	3.90	15.62	15.62	7.81	7.81	62.50	62.50	31.25	31.25
*P. vulgaris*	15.62	15.62	7.81	7.81	62.50	62.50	31.25	31.25	250	250	125	125
*C. albicans*	7.81	7.81	3.90	3.90	15.62	15.62	7.81	7.81	125	125	62.50	62.50
*A. brasiliensis*	7.81	7.81	3.90	3.90	15.62	15.62	7.81	7.81	125	125	62.50	62.50
*B. subtilis*	7.81	7.81	3.90	3.90	15.62	15.62	7.81	7.81	62.50	62.50	31.25	31.25

*P. aeruginosa, Pseudomonas aeruginosa ATCC 15442; S. aureus, Staphylococcus aureus ATCC 6538; E. hirae, Enterococcus hirae ATCC 10541; E. coli, Escherichia coli ATCC 10536; P. vulgaris, Proteus vulgaris ATCC 13315; C. albicans, Candida albicans ATCC 10231; A. brasiliensis, Aspergillus brasiliensis ATCC 16404; B. subtilis, Bacillus subtilis ATCC 6633; C_1_, Compound disinfectant 1; C_2_, Compound disinfectant 2; and Monomer, N-dodecyl-2-(piridin-1-ium)acetamide chloride (C_12_cmpCl)*.

#### Organic Matter Protection Test Results

In order to demonstrate that the bactericidal activity of the compound disinfectant is not affected by organic matter, an organic matter protection test was carried out. The compound disinfectant C_1_ increased the concentration of *C. albicans* and *B. subtilis* by 2-fold within 1min of action time. In the monomeric compound groups, the concentration of *S. aureus*, *E. hirae*, *E. coli*, *C. albicans*, and *B. subtilis* was also increased by 2-fold within 1min of action time, while there was no change in the other groups. These results indicated that the compound disinfectant was not affected by organic matter (Table 6).

**Table 6:** Organic matter protection test results.

Microorganism	C_1_ (mg/L)				C_2_ (mg/L)				Monomer (mg/L)			
	1min	5min	15min	30min	1min	5min	15min	30min	1min	5min	15min	30min
*P. aeruginosa*	31.25	31.25	15.62	15.62	62.50	62.50	31.25	31.25	250	250	125	125
*S. aureus*	3.90	1.95	1.95	0.97	15.62	15.62	7.81	7.81	125	62.50	31.25	31.25
*E. hirae*	7.81	7.81	3.90	3.90	15.62	15.62	7.81	7.81	125	62.50	31.25	31.25
*E. coli*	7.81	7.81	3.90	3.90	15.62	15.62	7.81	7.81	125	62.50	31.25	31.25
*P. vulgaris*	15.62	15.62	7.81	7.81	62.50	62.50	31.25	31.25	250	250	125	125
*C. albicans*	15.62	7.81	3.90	3.90	15.62	15.62	7.81	7.81	250	125	62.50	62.50
*A. brasiliensis*	7.81	7.81	3.90	3.90	15.62	15.62	7.81	7.81	125	125	62.50	62.50
*B. subtilis*	15.62	7.81	3.90	3.90	15.62	15.62	7.81	7.81	125	125	62.50	62.50

*P. aeruginosa, Pseudomonas aeruginosa ATCC 15442; S. aureus, Staphylococcus aureus ATCC 6538; E. hirae, Enterococcus hirae ATCC 10541; E. coli, Escherichia coli ATCC 10536; P. vulgaris, Proteus vulgaris ATCC 13315; C. albicans, Candida albicans ATCC 10231; A. brasiliensis, Aspergillus brasiliensis ATCC 16404; B. subtilis, Bacillus subtilis ATCC 6633; C_1_, Compound disinfectant 1; C_2_, Compound disinfectant 2; and Monomer, N-dodecyl-2-(piridin-1-ium)acetamide chloride (C_12_cmpCl)*.

### Carrier Test Results

Before conducting clinical trials in the field, we first established that the germicidal effect of the compound disinfectant on the carrier is the same or similar to the time-based germicidal test (time kill assay) before conducting clinical tests. As the compound is mainly used for air and ground disinfection in poultry farms, carrier tests were carried out on stainless steel sheets in accordance with the relevant standards. The results showed that the bactericidal activity of the compound disinfectants to *B. subtilis* and *A. brasiliensis* at 1min was slightly reduced compared to the time kill assay. This may be due to the fact that *molds* and *bacillus* are difficult to be killed, and the various environmental factors during the carrier test affect their bactericidal effect. However, the bactericidal effect increased significantly with time, indicating that contact time has an effect on the bactericidal activity. The monomer disinfectant control group was not as sensitive as the compound disinfectants group (Table 7). These results indicated that the carrier test was similar to the results of the time kill assay. Therefore, subsequent animal experiment was performed to determine its clinical bactericidal effect.

**Table 7:** Bactericidal concentrations resulting in a≥5 logarithmic reduction in the carrier quantitative suspension test.

Microorganism	C_1_ (mg/l)				C_2_ (mg/l)				Monomer (mg/l)			
	1min	5min	15min	30min	1min	5min	15min	30min	1min	5min	15min	30min
*P. aeruginosa*	31.25	31.25	15.62	15.62	62.50	62.50	31.25	31.25	500	250	125	125
*S. aureus*	3.90	1.95	1.95	0.97	15.62	15.62	7.81	7.81	62.50	62.50	31.25	31.25
*E. hirae*	7.81	7.81	3.90	3.90	15.62	15.62	7.81	7.81	125	62.50	31.25	31.25
*E. coli*	7.81	7.81	3.90	3.90	15.62	15.62	7.81	7.81	125	62.50	31.25	31.25
*P. vulgaris*	15.62	15.62	7.81	7.81	62.50	62.50	31.25	31.25	500	250	125	125
*C. albicans*	7.81	7.81	3.90	3.90	15.62	15.62	7.81	7.81	250	125	62.50	62.50
*A. brasiliensis*	15.62	7.81	3.90	3.90	31.25	15.62	7.81	7.81	500	125	62.50	62.50
*B. subtilis*	15.62	7.81	3.90	3.90	31.25	15.62	7.81	7.81	125	62.50	31.25	31.25

*P. aeruginosa, Pseudomonas aeruginosa ATCC 15442; S. aureus, Staphylococcus aureus ATCC 6538; E. hirae, Enterococcus hirae ATCC 10541; E. coli, Escherichia coli ATCC 10536; P. vulgaris, Proteus vulgaris ATCC 13315; C. albicans, Candida albicans ATCC 10231; A. brasiliensis, Aspergillus brasiliensis ATCC 16404; B. subtilis, Bacillus subtilis ATCC 6633; C_1_, Compound disinfectant 1; C_2_, Compound disinfectant 2; and Monomer, N-dodecyl-2-(piridin-1-ium)acetamide chloride (C_12_cmpCl)*.

### Analysis of Quaternary Ammonium Salt Resistance Genes

Disinfectants have been used to kill harmful microorganisms in the environment since their discovery, but misuse of disinfectants can cause microbial tolerance to disinfectants and reduction in the disinfectant killing effectiveness. We designed five pairs of quaternary-specific primers for disinfectant resistance-related genes and analyzed the presence of these genes in pathogenic bacteria to understand their resistance status. The results showed that none of the five quaternary ammonium resistance-associated genes (*qac E∆1, qac A/B, qac C, qac G, and qac J*) were present in the test strains, indicating that the test strains were less likely to develop resistance to the quaternary ammonium.

### Bacterial Reverse Mutation (Ames) Test Results

*S. typhimurium* histidine nutrient-deficient (*his^−^*) strains TA97, TA98, TA100, TA1535, and TA1537 were selected for Ames test using 6-well plates in the presence/absence of S9 metabolic activation, respectively. The number of responding colonies in each well was counted, and the mean value was obtained for each concentration group. The results showed that under non-metabolic activation condition, the number of colonies in the C_1_ (25ng/l and 50ng/l) group was 2-fold higher than that in the solvent control group for TA98 strain, but not for TA1535, TA97, TA1535, and TA1537. Similar results were also observed for C_2_ at the concentrations of 6.25 and 25ng/l, but not at the concentration of 50ng/l. Under metabolic activation conditions, the number of colonies in the C_1_ group at the concentration of 3.125–25ng/l (but not 50ng/l) was 2-fold higher than that in the solvent control group for TA98 strain. Similar results were also observed for C_2_ at the concentrations of 3.125–12.5ng/l, but not at the concentration of 25ng/l. Although the number of colonies for the combined disinfectants at all concentrations was 2-fold higher than that in the solvent control group for TA98 and TA97 strains, they did not reach 3-fold higher for TA1537 and TA1535 strains. Importantly, the number of responding colonies was not dose-dependent, indicating a negative result for Ames test for the disinfectants used in this study (Tables 8 and 9).

**Table 8:** Results of 48h bacterial reversion mutation under non-metabolic activation conditions (mean±SD, *n* =3).

Test substance	Concentration	Bacterial reversion colony count/well				
		TA97	TA98	TA100	TA1535	TA1537
ddH_2_O	20μg/ml	5 ± 2	2 ± 2	10 ± 6	2 ± 3	2 ± 0
C_1_	3.125ng/l	7 ± 2	3 ± 1	11 ± 9	2 ± 0	1 ± 2
	6.25ng/l	3 ± 6	2 ± 2	7 ± 4	2 ± 0	1 ± 0
	12.5ng/l	5 ± 1	3 ± 2	11 ± 1	1 ± 0	1 ± 1
	25ng/l	7 ± 1	5 ± 1	7 ± 5	2 ± 0	2 ± 1
	50ng/l	2 ± 3	5 ± 0	6 ± 10	2 ± 2	1 ± 1
C_2_	3.125ng/l	4 ± 12	2 ± 6	7 ± 7	1 ± 2	1 ± 1
	6.25ng/l	6 ± 7	4 ± 1	11 ± 9	4 ± 5	1 ± 0
	12.5ng/l	8 ± 2	3 ± 2	7 ± 11	2 ± 1	1 ± 2
	25ng/l	3 ± 4	4 ± 1	7 ± 5	2 ± 2	1 ± 0
	50ng/l	4 ± 1	2 ± 2	5 ± 3	1 ± 1	1 ± 0
Positive control	Name	AF-2	AF-2	AF-2	NaN_3_	9-AA
	Concentration	1μg/ml	10μg/ml	1μg/ml	25μg/ml	2000μg/ml
	Results	76 ± 6^***^	42 ± 10^***^	91 ± 5^***^	72 ± 7^***^	70 ± 10^***^

C_1_, Compound disinfectant 1; C_2_, Compound disinfectant 2; ddH_2_O, Sterilized distilled water; Positive control, 2-(2-furyl)-3-(5-nitro-2-furyl)acrylamide; NaN_3_ (sodium azide); 9-AA (9-aminoacridine); ^***^p<0.001; ^**^p<0.01; and ^*^p<0.05.

**Table 9:** Results of 48h bacterial reversion mutagenesis under metabolic activation conditions (+ rat S9; mean±SD, *n* =3).

Test substance	Concentration	Bacterial reversion colony count/well				
		TA97	TA98	TA100	TA1535	TA1537
ddH_2_O	20μg/ml	5 ± 2	2 ± 3	10 ± 6	2 ± 3	2 ± 0
C_1_	3.125ng/l	7 ± 2	5 ± 5	5 ± 4	4 ± 0	3 ± 0
	6.25ng/l	6 ± 9	4 ± 2	4 ± 7	2 ± 1	2 ± 1
	12.5ng/l	4 ± 9	5 ± 4	10 ± 3	1 ± 4	1 ± 0
	25ng/l	8 ± 8	5 ± 1	6 ± 2	1 ± 0	2 ± 1
	50ng/l	4 ± 11	2 ± 2	8 ± 3	2 ± 1	2 ± 1
C_2_	3.125ng/l	6 ± 6	5 ± 2	7 ± 6	4 ± 0	2 ± 1
	6.25ng/l	7 ± 1	5 ± 8	6 ± 4	1 ± 2	1 ± 2
	12.5ng/l	11 ± 4	7 ± 4	5 ± 5	1 ± 0	2 ± 0
	25ng/l	5 ± 4	2 ± 6	12 ± 4	1 ± 1	1 ± 1
	50ng/l	4 ± 0	4 ± 4	8 ± 2	1 ± 3	1 ± 0
Positive control	Name	2-AA	2-AA	2-AA	2-AA	2-AA
	Concentration	33μg/ml	33μg/ml	33μg/ml	100μg/ml	100μg/ml
	Results	81 ± 6^***^	88^***^	85 ± 7^***^	15 ± 20^**^	17 ± 12^**^

C_1_, Compound disinfectant 1; C_2_, Compound disinfectant 2; ddH_2_O, Sterilized distilled water; Positive control, 2-AA (2-Aminoanthracene); ^***^p<0.001; ^**^p<0.01; and ^*^p<0.05.

### Toxicological Analysis of Compound Disinfectants

#### Acute Oral Toxicity Results

**Figure 1:**
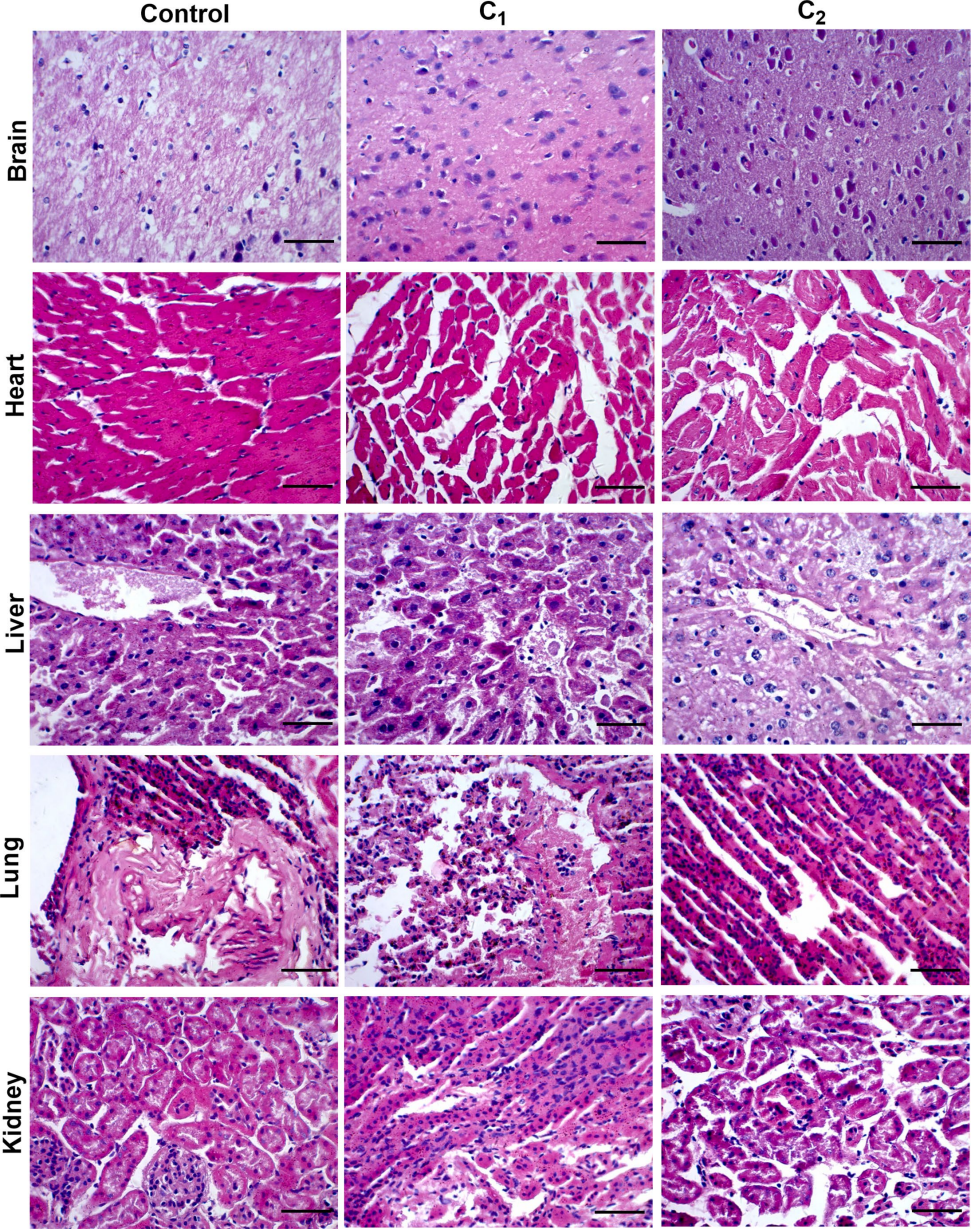
Results of pathological tissue section: Hematoxylin staining. C_1_, Compound disinfectant 1; C_2_, Compound disinfectant 2. Bar=20μm.

Acute oral toxicity tests were carried out with reference to European standards. Two compound disinfectants had LD_50_ >5,000mg/kg in mice and LD_50_ >2000mg/kg in chicks. Pathological tissue findings showed that there were no abnormal changes within the tissues of brain, liver, heart, lung, and kidney in the experimental group comparing to the control group, indicating that the compound disinfectants belong to the actual non-toxic grade (Figure 1).

#### Results of DNA Damage in Chicks

**Figure 2:**
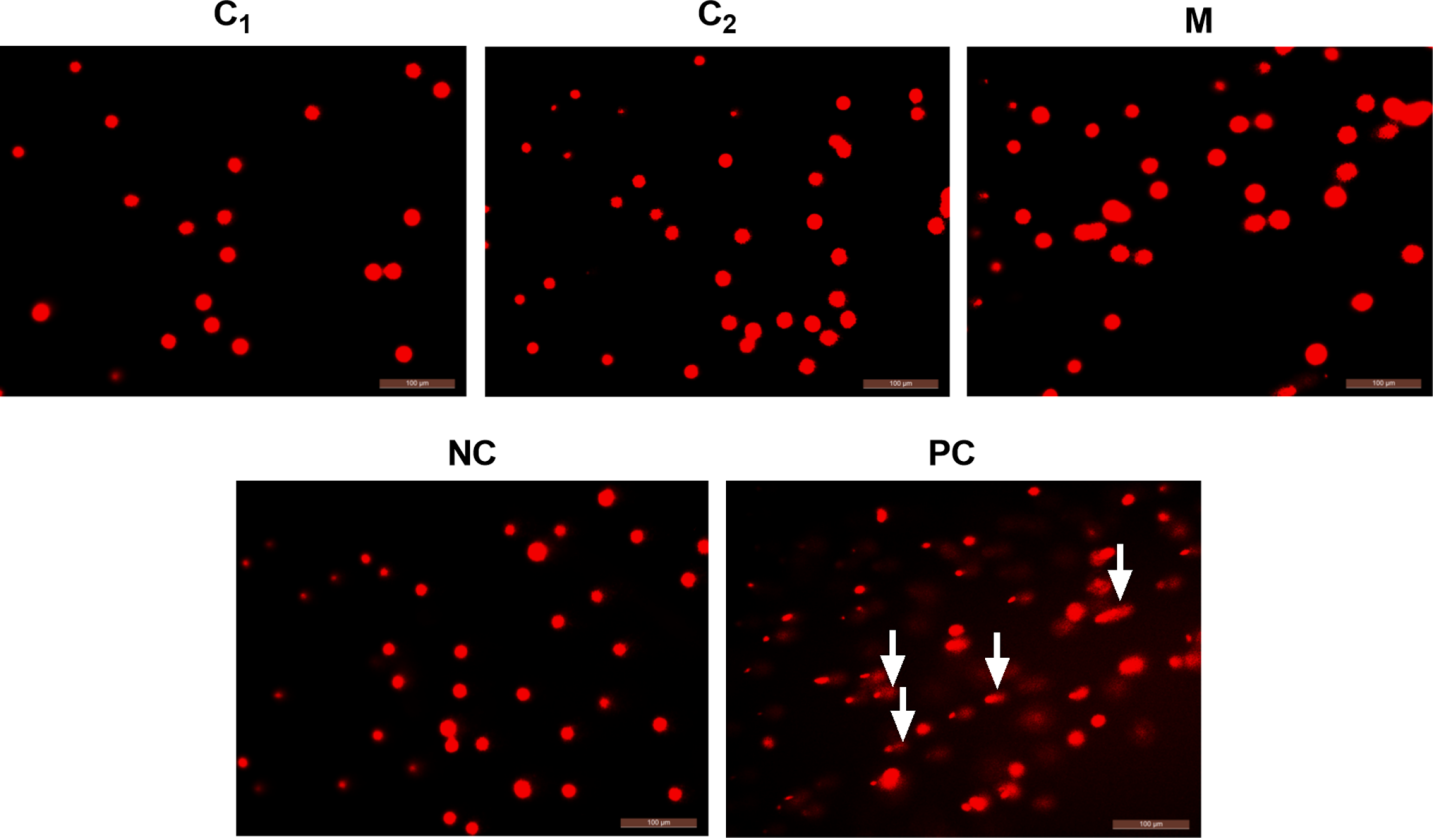
Results of *in vivo* single-cell gel electrophoresis assay. C_1_, Compound disinfectant 1; C_2_, Compound disinfectant 2; M, *N*-dodecyl-2-(piridin-1-ium)acetamide chloride (C_12_cmpCl); NC, Negative control; and PC, Positive control. The arrow indicates the comet’s trailing tail.

After the oral toxicity test, the chicks were dissected and the liver, spleen, and thymus were collected to determine the DNA damage by comet electrophoresis test. The stained DNA samples were observed under a fluorescence microscope, and the undamaged cells showed a round fluorescent core, i.e., a comet head, without a tail. Damaged cells, on the other hand, have comet tails reaching toward the anode and forming a bright head and tail. Photographs were taken with a fluorescent inverted microscope, and at least 100 cells in each sample were randomly selected for determination. The cells were characterized according to the ratio of the amount of DNA in the trailing tail to the total DNA in the cells. The degree of DNA damage was classified into five levels: level 0 (no damage, normal cells, <5%); level 1 (mild damage, 5–20%); level 2 (moderate damage, 20–40%); level 3 (high damage, 40–95%); and level 4 (severe damage, >95%). The results showed that most of the cells in the positive control group were lysed and had typical trailing phenomenon. In contrast, in the negative control group and disinfectant groups, most of the cells had clear nuclei without trailing phenomenon (Figure 2).

### Bacteriostasis Effect of Compound Disinfectant on Strains From Different Sources

To demonstrate the bactericidal ability, we further tested the combined disinfectants on 10 clinical isolates. The results show that there was no significant difference of bacteriostasis effect among groups of strains from different sources (*p*>0.05). Two compound disinfectants also had strong effects on Gram-positive bacteria, and they immediately killed most pathogenic bacteria within 10min of action. These results indicate that the compound disinfectants have a stable antibacterial effect on strains from different sources and provide the basis for further clinical application (not shown in the text).

### Application of the Two Compound Disinfectants in Clinical Settings

**Figure 3:**
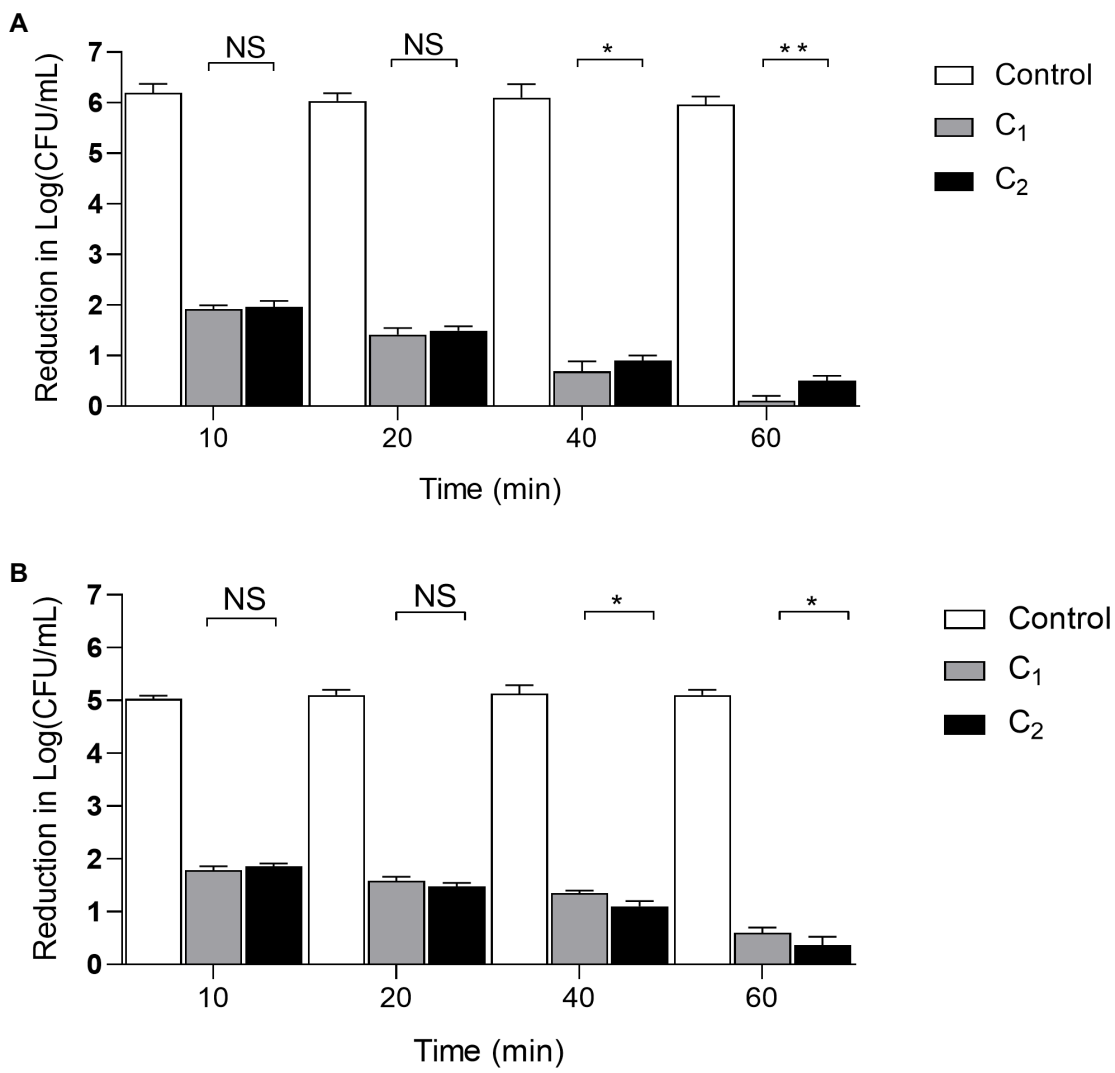
The disinfection effect of Compound disinfectant. Clinical trials were divided into disinfection group (C_1_, C_2_) and control group. Four different action times were selected to determine the best action time and the clinical bactericidal effect of the disinfectant according to the logarithmic value of the microbial reduction between each group. **(A)**: Air disinfection; **(B)**: Ground disinfection; ***p< 0.001; **p< 0.01; *p< 0.05, NS: Insignificant difference, *n*=3. Data were presented as mean±SD; C_1_, Compound disinfectant 1; and C_2_, Compound disinfectant 2.

We examined the disinfection of the air and floor of the chicken farm after 10, 20, 40, and 60min of treatment. The results showed that when the action time of the compound disinfectants reached 10min, a good disinfection effect can be achieved. As the action time was extended, the disinfection effect was gradually increased. When the action time was 40min, C_1_ had a better disinfection effect on air and ground than C_2_ (*p* <0.05). When the action time was extended to 60min, the difference between C_1_ and C_2_ increased (*p* <0.01). This indicates that with the increase of time, C_1_ has a better disinfection effect on the air and can be used for the disinfection of air in chicken farms, while C_2_ can be used for disinfection of ground in chicken farms. The difference between C_1_ and C_2_ was increased with time (Figure 3).

Furthermore, an increase in temperature promotes the activity of the compound disinfectant. During the clinical trials in the field, the results were greatly influenced by the cold weather and the incomplete removal of dirt from the chicken coop. Under laboratory conditions, the bactericidal effect was stable at a fixed temperature, and the bactericidal effect increased as the temperature increased.

## Discussion

With our increasing demand for poultry products, the poultry industry has expanded rapidly. However, due to environmental factors, e.g., excessive feeding density, excessive humidity, and unreasonable light, it is easy to cause serious respiratory diseases, affect weight gain and feed utilization, reduced egg production rate, carcass quality degradation, and even resulted in a considerable number of deaths, which decreases the economic benefits of poultry farming. The environmental conditions of the poultry house have a significant impact on the respiratory mucosa of poultry. A good environment condition can facilitate the healthy growth of chickens and reduce the occurrence of respiratory diseases. Disinfectant can kill pathogenic microorganisms and provide poultry a good growing environment. Therefore, choosing the right disinfectant is the key factor for the success of disinfection. Quaternate ammonium salt is a cationic surfactant that has good bactericidal properties with mild and non-irritating advantages (Faria et al., 2018). If the quaternate ammonium disinfectant is prepared with 70% alcohol, the penetration effect can be significantly increased. For this reason, this study synthesized a quaternate ammonium salt and formulated a new type of broad-spectrum disinfectant based on the combination of this quaternate ammonium salt with other disinfectants in order to overcome the disadvantages, e.g., drug resistance, drug residues, and irritation. Respiratory diseases in poultry are mainly caused by *Streptococcus*, *Staphylococcus*, *E. coli*, and *Pasteurella*. We evaluated the antibacterial and antifungal activity of two kinds of quaternary ammonium compound disinfectants against these pathogens. The results showed that the compound disinfectants had stronger bactericidal effect compared to previous studies (Kuda et al., 2008). The two compound disinfectants have good antibacterial and bactericidal effects on *Streptococcus* and *Staphylococcus*. In addition, the compound disinfectant has a bactericidal effect not only on common microorganisms, but also on *P. aeruginosa*, *B. subtilis*, *C. albicans*, and fungi. The shortest bactericidal effect time of the two compound disinfectants was 1min, and the bactericidal efficacy was more than 99.90% against the indicator bacteria. It should also be noted that the same farm should not use the same disinfectant for a long time. The incidence of drug resistance can be greatly reduced when a different type of disinfectant was used. The quaternary ammonium salt surfactant used in this study has a new structure and has not been described so far. Thus, this compound belongs to a new type of disinfectant, which could potentially reduce the risk of resistance compared to the disinfectants that are commercially available. To further demonstrate that the compound does not induce resistance and mutagenicity, we analyzed the resistance genes and performed bacterial reversion mutation tests using standard strains TA97, TA98, TA100, TA1535, and TA1537. The results showed that the two compound disinfectants were not mutagenic in the experimental dose range. Moreover, five genes associated with quaternary ammonium salt resistance were not observed in the test strains, indicating that resistance is less likely to be induced by the compound disinfectants.

In this study, an acute oral toxicity test was conducted on mice to determine its safety under laboratory conditions. The results showed the compound disinfectants had no observable signs of toxicity in mice. At the same time, we conducted acute oral toxicity tests on chicks and DNA damage tests on chicks, and the results showed that both compound disinfectants were not toxic to chicks, and there was no DNA damage. Moreover, the compound disinfectants were effective against strains of different sources, indicating that they can be potentially applied for the disinfection of poultry farms. In clinical application, Begec et al. (2013) studied the antibacterial effect of ketamine combined with propofol *in vitro*. Kuda et al. (2008) tested the bactericidal effect of benzalkonium chloride on the surface of an object. In the carrier assay, Kuda et al. chose stainless steel as the carrier to measure the number of colonies falling on the stainless-steel carrier attacked by *E. coli* and *Staphylococcus*. In this study, the maximum concentrations of the two compound disinfectants to achieve bactericidal effect on *E. coli*, *P. aeruginosa*, *S. aureus*, *B. subtilis*, *C. albicans*, *P. vulgaris*, *E. hirae*, and *A. brasiliensis* on the surface of stainless-steel carriers (1cm) were lower than the concentration of other disinfectants as reported previously (Kuda et al., 2008), indicating that the compound disinfectants were more effective. In clinical applications, we applied these two compound disinfectants to the air and ground of the poultry farm. The bactericidal effect increases with the increase of the action time, indicating a promising prospect of its future application in poultry farms.

The ideal disinfectant should have a good bactericidal effect, good stability and low irritation, and are cheap, convenient, and safe for humans and animals (Burgess et al., 2017; West et al., 2018). However, disinfectant products that are currently available cannot meet this standard. Therefore, in this study, we synthesized a new type of disinfectant. The disinfectant is composed of a new surfactant C_12_cmpCl that were used as the monomer active substances for bactericidal activity tests and chlorhexidine acetate or glutaraldehyde at a certain ratio. This compounding method can not only significantly enhance the bactericidal capacity of chlorhexidine acetate and glutaraldehyde but also reduce the concentration of cationic surfactant C_12_cmpCl. In addition, the new disinfectant is less likely to develop resistance and is not mutagenic. The new disinfectant is non-toxic and non-irritating, and has a good bactericidal effect on different types of bacteria from different sources. Although glutaraldehyde itself has certain irritation or toxic effect, such effect is greatly reduced after it is combined with quaternary ammonium salt. The compound disinfectant (C_2_) can be applied for the floor cleaning and disinfection of poultry houses.

In conclusion, the new compound disinfectant developed in this study is suitable for air and ground disinfection in poultry farms. It has the characteristics of a novel structure, low irritation, and good safety, and most pathogenic bacteria can be killed within 10min. The new compound disinfectant can significantly reduce costs, improve safety, and provide a good prospect for clinical application. Therefore, it is broadly applicable in controlling poultry diseases and promoting the healthy development of animal husbandry.

## Data Availability Statement

The original contributions presented in the study are included in the article/supplementary material; further inquiries can be directed to the corresponding author.

## Author Contributions

ZZ and LY contributed to the conception and design of this work. NC, PQ, and LJ participated in sample collection, laboratory experiments, and data analysis. YY and HW completed Ames trial. LJ synthesized the compound in the pre-experimental stage. NC and ZZ drafted the manuscript. All authors read and approved the final manuscript.

## Funding

This study was supported by the National Science and Technology Ministry (2013BAD21B01–1), Open Fund Project of Heilongjiang Provincial Key Laboratory of Fine Chemicals (2012–2014), and Heilongjiang Bayi Agricultural University Support Program for San Heng San Zong (TDJH202002).

## Conflict of Interest

The authors declared no potential conflicts of interest with respect to the research, authorship, and/or publication of this article.

## Publisher’s Note

All claims expressed in this article are solely those of the authors and do not necessarily represent those of their affiliated organizations, or those of the publisher, the editors and the reviewers. Any product that may be evaluated in this article, or claim that may be made by its manufacturer, is not guaranteed or endorsed by the publisher.
